# Follicular lymphoma manifests as multiple erosive and proliferative lesions of the oral mucosa: case report and brief literature review

**DOI:** 10.1186/s12903-022-02567-7

**Published:** 2022-11-19

**Authors:** Yuqi Wu, You Zhang, Chunyu Li, Yulang Xie, Sixin Jiang, Yuchen Jiang, Yan Qiu, Xiaobo Luo, Qianming Chen

**Affiliations:** 1 State Key Laboratory of Oral Diseases, National Clinical Research Center for Oral Diseases, Research Unit of Oral Carcinogenesis and Management, Chinese Academy of Medical Sciences, West China Hospital of Stomatology, Sichuan University, 610041 Chengdu, China; 2 Department of Pathology, West China Hospital, Sichuan University, Chengdu, 610041 China

**Keywords:** Follicular lymphoma, Erosive and proliferative lesions, Oral manifestation, Case report

## Abstract

**Background:**

Erosion is one of the most common and basic lesions of oral mucosal diseases. Long-term refractory oral erosions, induced by autoimmune blistering diseases, infectious diseases, malignant diseases, and some rare conditions, may substantially reduce the quality of life of patients or even constitute a life-threatening condition, resulting in a clinical dilemma regarding the accurate diagnosis and precise management of these diseases. As a special type of malignant lymphoma, most lesions of follicular lymphoma (FL) in the oral mucosa present as masses or swelling of the oral mucosa, while emerging novel presentations lead to intractable diagnoses. Hence, diagnostic algorithms for such diseases are clinically required.

**Case presentation:**

A 55-year-old female patient presented to the clinic with long-lasting oral mucosal erosions and proliferative lesions. Blood tests, pathological examinations of oral lesions including haematoxylin–eosin (HE) staining, and direct immunofluorescence precluded all of the potential diagnoses described previously. Unexpectedly, positron emission tomography/computed tomography (PET/CT) and abdominal CT of the patient revealed a dense mass in the retroperitoneal area, and the final diagnosis of the retroperitoneal mass was FL. After three courses of chemotherapy conducted by the haematologist, the erosion and proliferative lesions in the patient's oral mucosa had significantly improved. HE and immunohistochemical staining results of intraoral lesions also confirmed it as oral FL. The successful diagnosis of FL in this case is of great clinical significance, as the oral and abdominal FL were treated in a timely manner to avoid unfavourable outcomes.

**Conclusions:**

To the best of our knowledge, this is the first case of FL that exhibited widespread erosions interspersed with proliferative lesions. Clinicians should be aware of oral FL or seek systemic factors in the presence of similar refractory oral erosions when treatment is non-responsive and the diagnosis is intractable.

## Background

Oral mucosal diseases are characterised by their high incidence and wide extent of involvement. For example, the prevalence of recurrent aphthous ulcer (RAU) ranges from 5 to 25% [1], and the associated Behcet’s disease might involve other body sites besides the oral mucosa [2]; thus, oral mucosal lesions may serve as an indicator of systemic diseases. Erosions or ulcers are common manifestations and basic lesions of oral mucosal diseases, with erosions histologically presenting as partial defects of the mucosal epithelium and ulcers as defects of the entire epithelial layer. Oral mucosal diseases such as oral lymphoma may manifest as erosions and ulcers [3]. Long-lasting erosions or ulcers may lead to severe distress and a significant decrease in quality of life, and in some cases, may even be life-threatening. For instance, long-term RAU or erosion poses a substantial threat to the quality of life of patients by causing difficulties with daily food intake or weight loss due to severe pain, repeated seeking of medical advice, and compelled consumption of various types of medicine including steroids, immunosuppressants, and even biologics such as intravenous immunoglobulins, thereby inducing serious physical and psychological pressure [4–6]. In addition, recalcitrant and complicated oral ulceration or erosions might represent a non-specific sign of malignancies with poor prognosis; for example, a one-month large palatal ulceration resistant to routine treatment was ultimately diagnosed as a presentation of NK/T-cell lymphoma, which has aggressive behavior and a poor outcome [7].

Apart from these common aetiologies contributing to oral erosions, such as oral lichen planus (OLP), a small proportion of persistent oral erosions may be oral manifestations of systemic diseases or other factors. In recent years, oral medicine clinicians and pathologists have faced emerging challenges owing to refractory and complicated oral erosions. First, regular inquiry of medical history and oral examination are insufficient to obtain the actual diagnosis of some challenging oral erosions. Second, the definite diagnosis of some non-specific oral signs may require biopsy, which must include normal-appearing tissue and have adequate depth in addition to diseased tissue, after which haematoxylin–eosin (HE), direct immunofluorescence (DIF), and immunohistochemistry (IHC) might be required to reveal the actual disease. Thus, the accuracy of the whole process is largely dependent on the experience of dental clinicians and oral pathologists. Third, besides the detection of biopsied lesions, some adjuvant examinations are recommended to identify systemic factors responsible for the oral erosions, encompassing computed tomography (CT), ultrasonography, blood testing, whole exome sequencing, and so forth. Finally, for refractory oral erosions that cannot be diagnosed through the aforementioned measures, multi-disciplinary team consultation, diagnostic treatment, and further biopsy may be required to achieve an adequate diagnosis [3, 7–9].

A large number of oral mucosal diseases can be characterised by oral mucosal erosion, including oral allergic diseases [10], oral infectious diseases such as tuberculosis (TB) [3], pemphigus/pemphigoid [11], paraneoplastic autoimmune multiorgan syndrome (PAMS) [12], oral potentially-malignant disorders (OPMD) represented by OLP [13], oral malignancies including oral squamous cell carcinoma (OSCC) [14], and genetic diseases such as dyskeratosis congenita [8] (Table 1). These diseases may be distinguished from each other based on various features, such as the clinical duration and medical history, and by engaging diagnostic approaches, including examination of biopsied tissue and systemic adjuvant examination.

**Table 1:** Differential diagnoses of long-lasting oral erosive lesions based on existing literatures

Diagnosis	Manifestation	Potential adjuvant examinations
**Autoimmune blistering diseases** [11, 15–17]	Bullous lesions, irregular erosion, hyperemic lesion, Nikolsky’ sign of oral mucosa and/or skin No systemic involvement of other organs	HE: subepithelial or intraepithelial bulla DIF: linear or reticular deposition of C3, IgG, IgA and IgM in the basement membrane or in the intraepithelial area
**PAMS/ PNP** [12, 18]	Erosions or bulla of the skin and mucous membrane accompanied by occult tumors	HE: loosening of spinous layer, keratinocyte necrosis, interface dermatitis DIF: deposition of C3, IgG and/or IgA and IgM in the intraepithelial area and basement membrane
**Infectious diseases**		
*** Syphilis*** [19, 20]	Secondary syphilis: round gray-white plaques, congested, diffusely flushed mucosa with erosions or ulcers	Blood test: non-syphilis spirochete antigen serologic test and specific syphilis spirochete antigen serologic test HE: endovasculitis
*** AIDS*** [21]	Oral candidiasis; Deep or recurrent oral ulcers; Other non-specific oral lesions	Blood test: HIV antibody
*** Oral tuberculosis*** [3, 22]	Hard nodules, long-lasting erosions or deep ulcers or with irregular margins in the oral mucosa	HE: typical Langerhans giant cells Positive result of acid-fast staining and PCR for TB DNA
**OPMD**		
*** OLP*** [13, 23, 24]	Erosions, typical symmetrical white striae	HE: hyperkeratosis, liquefied degeneration, and infiltration of band-like lymphocytes
*** OLK*** [25]	White plaques with erosive and/or ulcerative lesions	HE: epithelial hyperplasia with hyper parakeratosis or hyper(ortho)keratosis, various degree of epithelial dysplasia
*** OE*** [25]	Demarcated and flat scarlet patches accompanied with erosions or ulcerations	HE: Epithelial atrophy and lack of stratum corneum with diverse degree of epithelial dysplasia
*** DLE*** [26]	Reddish area with central atrophy and depression with concomitant erosions	HE: Liquefaction of basal cells and perivascular lymphocytic infiltration, epithelial atrophy and lack of stratum corneum
**OSCC** [14]	Localized ulcer and erosions with firm texture	HE: presence of typical squamous cell carcinoma
**PSV** [27, 28]	Proliferative pustular lesions and subsequent erosions in the oral mucosa	HE: acanthosis, intraepithelial and subepithelial micro abscesses, accompanied by infiltration of neutrophils and eosinophils Colonoscopy: inflammatory bowel disease
**LCH** [29, 30]	Ulcers or erosions with inflammatory reddened margins and tenderness	HE: eosinophilic granuloma and LCH cells IHC: CD1a or S100 or Langerin positive CT: cranial or maxillofacial bone abnormalities
**Dyskeratosis congenita** [8]	Mucosal leukoplakia, persistent oral mucosal erosions	Whole exome sequencing HE: non-specific inflammation Bone marrow aspiration: aplastic anemia

*Abbreviations: HE* hematoxylin–eosin, *DIF* direct immunofluorescence, *PAMS* paraneoplastic autoimmune multiorgan syndrome, *PNP* paraneoplastic pemphigus, *AIDS* acquired immune deficiency syndrome, *HIV* human immunodeficiency virus, *TB* tuberculosis, *PCR* polymerase chain reaction, *OPMD* oral potentially malignant disorders, *OLP* oral lichen planus, *OLK* oral leukoplakia, *OE* oral erythroplakia, *DLE* discoid lupus erythematosus, *OSCC* oral squamous cell carcinoma, *PSV*: pyostomatitis vegetans, *LCH* Langerhans cell histiocytosis, *CT* computed tomography, *IHC* immunohistochemistry

We recently reported a woman with multiple refractory and scattered oral erosions accompanied by several proliferative and nodule-like lesions who had failed to respond to conventional treatment regimens (low-dose oral prednisone with dexamethasone mouthwash) and was finally diagnosed with oral manifestations associated with abdominal follicular lymphoma (FL) after a multidisciplinary consultation. FL is a lymphoid tissue systemic malignancy that exhibits germinal center B B-cell differentiation, and a small proportion of patients with FL might have poor outcomes [31]. Non-Hodgkin’s lymphomas (NHL) is a special type of lymphoma which frequently involves the head and neck region, and about 20–25% of NHL are diagnosed as FL [31]. Orally, FL typically presents as masses or swelling of the oral mucosa, which is a non-specific presentation among oral mucosal diseases, thus triggering difficulties and complexities in diagnosis [32]. The final diagnosis of this case was made through a combination of the clinical manifestations, thorough medical history enquiry, radiographic examination, multi-disciplinary cooperation, and pathological findings, exemplifying the diagnostic logistics of such complicated cases. To the best of our knowledge, this is the first case report of secondary FL presenting as widespread erosive and proliferative lesions in the oral mucosa, which might serve as a significant reminder in our future clinical practice.

## Case presentation

**Fig. 1:**
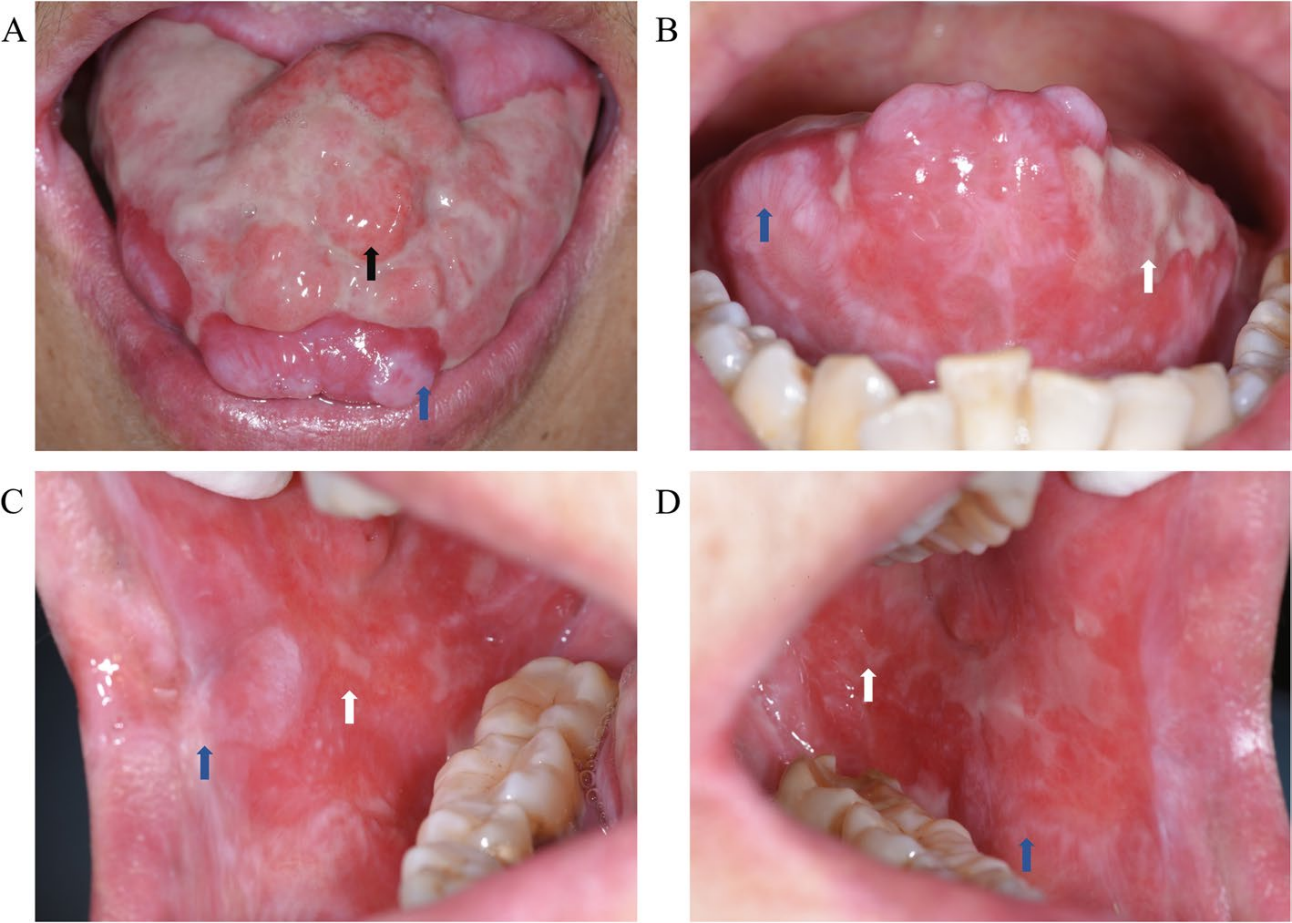
Oral mucosal manifestations of the patient. Widespread erosive lesions on the dorsum of tongue, interspersed with several mucosal proliferative or nodule-like lesions (**A**). Linear or reticular white striae with erosions and hyperemia could be observed on bilateral tongue margins (**B**), right angle of the mouth (**C**), and the lower part of right and left buccal mucosa (**C** and **D**). Black arrows: proliferative lesions with erosive surface. Blue arrows: reticular white striae. White arrows: erosions. All images were captured at the resolution of 300 dpi

A 55-year-old woman presented to our oral medicine clinic with refractory and recurrent erosions of the oral mucosa persisting for more than 2 years. Her condition had gradually worsened over the past 6 months, with almost no healing period for the oral erosions. The patient complained of severe and obvious pain after eating irritating food. Upon clinical inspection, a region of erosion with a surface area of 40 mm × 20 mm was observed on the dorsum of the tongue, interspersed with several proliferative or nodule-like lesions with diameters ranging from 4 to 6 mm. The height of nodules was about 5 mm above the mucosal level, which was palpated and considered to be of moderate texture (Fig. 1A). Linear or reticular white striae accompanied by erosions and hyperaemia were observed on the right angle of the mouth, inner mucosa of both lips, bilateral tongue margins, and the lower part of the buccal mucosa (Fig. 1B, C, and D). The whole gingiva was hyperaemic and eroded along with shallow vesicles. The Nikolsky sign was positive, and the tongue was flexible and soft, which is the same for typical oral mucosa. Overall, periodontal hygiene was poor. In addition, enlarged lymph nodes were palpated in both mandibular regions, and the lymph nodes in the left mandibular region were relatively fixed without pain upon touching. A history of hypertension and a surgery for total hysterectomy were recorded upon medical history enquiry, and no drug allergy was revealed.

In terms of potential diagnosis, given the atypically widespread and refractory erosions on the oral mucosa, autoimmune blistering diseases, special infections such as human immunodeficiency virus (HIV) and syphilis, OLP, or malignant lesions, came up as the initial impression based on our clinical experience. Next, several examinations were suggested to achieve the diagnosis, including routine blood tests, glucose tests, biochemical tests, bullous disease antibodies against Dsg1, Dsg3, and Bp180, and testing for HIV and syphilis; negative findings were indicated in these tests. Furthermore, chest computed tomography (CT), abdominal ultrasonography, and biopsy of the inner mucosal lesions of the right upper lip were performed.

**Fig. 2:**
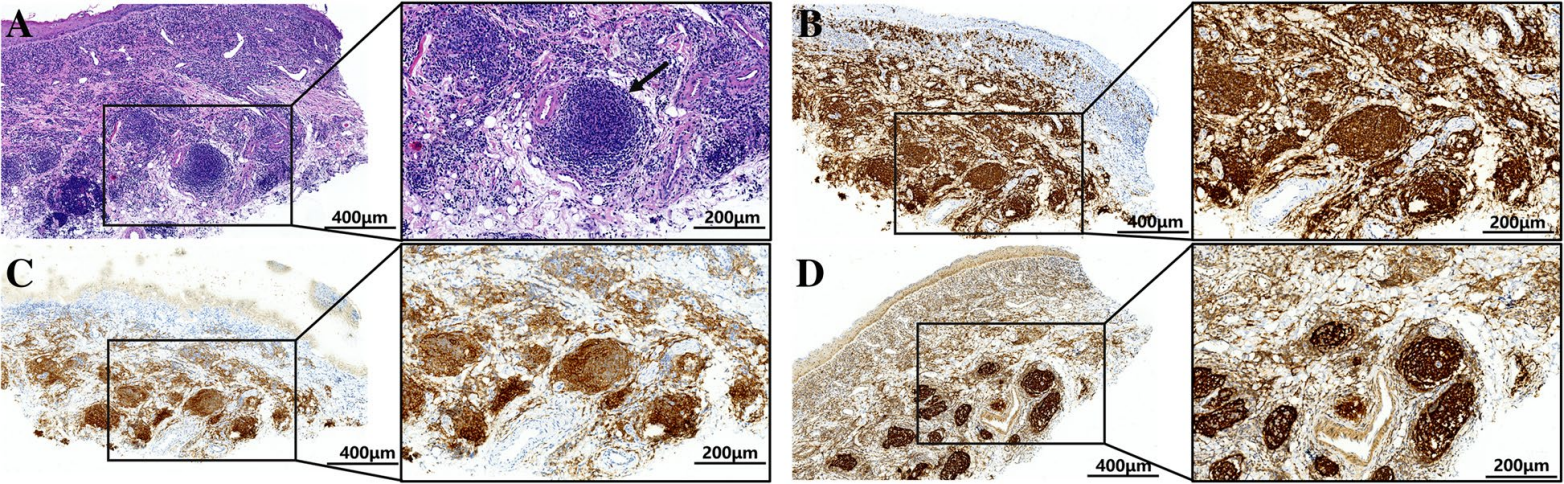
Pathological examination of the oral lesion. **A** HE staining of the right upper lip mucosa showed nodular aggregation of lymphocytes and plasma cells in the subepithelial connective tissue and around the blood vessels. **B-D** IHC result of the right upper lip mucosa showed follicular-like structures presenting as CD20 ( +), BCL-2 ( +), CD21 ( +), respectively. Black arrow: nodular aggregation of lymphocytes. All stained sections were imaged using Aperio Versa (Leica, Germany) at 100 × and 200 × magnification, and the images of stained slides were acquired at the resolution of 300 dpi. Abbreviations: HE: hematoxylin–eosin; IHC: immunohistochemistry; BCL-2: B-cell lymphoma-2

Meanwhile, the patient required medication during the process of receiving further tests. This course included 30 mg of prednisone per day for one week, 50 mg of thalidomide per day for 10 days and 0.1 mg/mL dexamethasone mouthwash; however, no significant recovery of the oral erosions and proliferative lesions was observed at the revisit, which emphasized the complexity of the disease and the potential systemic nature. For the biopsy, the inner-side mucosa of the right upper lip was selected because of the co-existence of erosion and white striae, which was not visibly present on the tongue. After HE staining, nodular aggregation of lymphocytes and plasma cells was observed in the subepithelial connective tissue and around the blood vessels under a microscope (Fig. 2A). The DIF test for diagnosing bullous disease showed negative results for IgA, IgG, IgM, and C3. In addition, chest CT was negative. However, positron emission tomography/computed tomography (PET/CT) of the patient, which was further suggested, provided a surprising clue. Specifically, the inspection showed increased metabolism in the salivary glands, an enlarged liver and spleen, and multiple hypermetabolic lymph nodes throughout the body. In addition, significantly thickened soft tissue was observed in the retroperitoneal area on abdominal ultrasonography, which encapsulated the abdominal aorta and mesenteric vessels, and an abundant blood supply was observed. All the above signs indicate haematologic disease in the retroperitoneal area. After visiting the haematology department, pathological examination of the patient's retroperitoneal mass revealed FL grade I-II and stage IV, and the patient was categorised into the intermediate-risk group, with the FL international prognostic index (FLIPI) score defined as 2. In order to investigate whether the intraoral lesion was related to lymphoma in the abdomen, the tissue sample from the upper lip mucosa was further stained by IHC, which showed follicular-like structures presenting as CD20 ( +) and B-cell lymphoma 2 (BCL-2) ( +), while the staining for CD10, CD5, and CD3 was negative, and CD21, CD23, and follicular dendritic cells (FDC) were positive (Fig. 2B-D). Thus, in light of the masses in the retroperitoneal area and HE together with IHC results of the oral tissue, the diagnosis of oral involvement by FL was finally made upon multidisciplinary discussion.

**Fig. 3:**
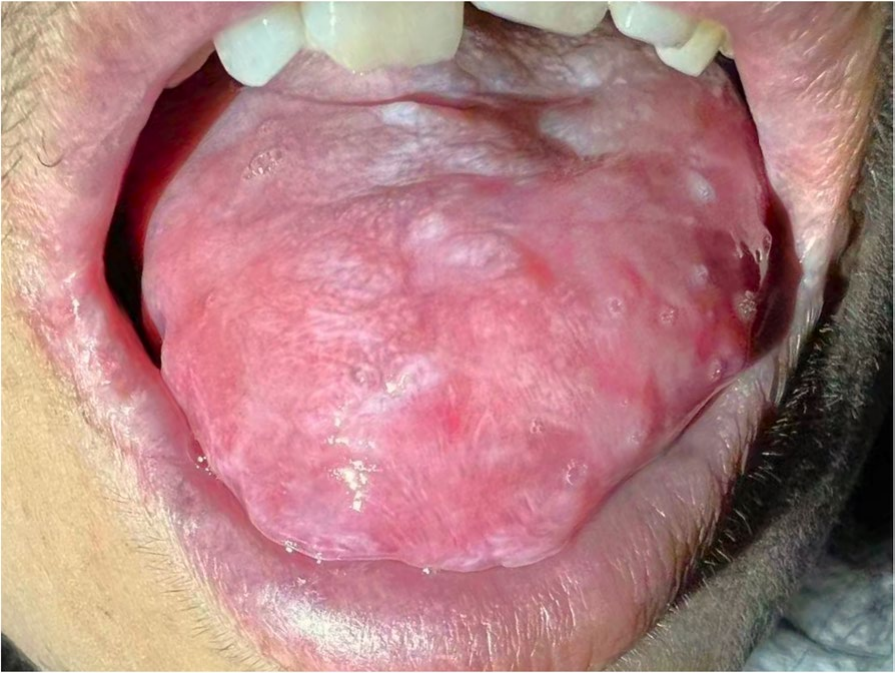
Recent follow-up imaging of the patient’s dorsum of tongue shows significant remission of the erosions and proliferative lesions after undergoing standardized chemotherapy against the follicular lymphoma. The image was captured at the resolution of 300 dpi

The patient was then referred to haematology department for further treatment of FL, and 0.1 mg/mL dexamethasone together with 2% sodium bicarbonate mouthwash was recommended for topical treatment of oral lesions. After meticulous evaluation by a haematologist, the patient was treated with a rituximab, cyclophosphamide, doxorubicin, vincristine, and prednisone (R-CHOP) regimen as follows: rituximab 500 mg D0, vinorelbine 30 mg D1, pirarubicin 60 mg D1, cyclophosphamide 1 g D1, hydrogenated prednisone 40 mg D1, D5. Over three courses of chemotherapy, the intraoral lesions improved significantly, the nodule-like lesions on the dorsum of the tongue disappeared, and the oral erosions were also controlled. The patient was then recommended routine visits to the haematology clinic every 2 months, and no further recurrence of oral lesions was observed in the follow-up visits until May 2022 (the third year after the onset of disease) (Fig. 3).

## Discussion and conclusions

Here, we report a case of long-lasting refractory erosions in the oral mucosa for more than 2 years, which was finally diagnosed as oral manifestations of FL. Although the diagnosis process had consumed tremendous time and effort, the patient received appropriate treatment.

As shown in Table 1, there are several common diseases in which long-term oral erosion might present as the main manifestation, including autoimmune blistering diseases such as pemphigus vulgaris and pemphigoid, acquired immune deficiency syndrome (AIDS), syphilis, OPMD such as OLP, and oral malignant lesions (Table 1). Autoimmune blistering diseases may present with bullous lesions in the oral mucosa, or manifest as large areas of irregular erosive and hyperaemic lesions following the rupture of the bulla [11, 15], the diagnosis of which is mainly dependent on HE and DIF results of lesions [16, 17]. To diagnose AIDS and syphilis, which may also present with non-specific oral erosions, blood tests for specific antibodies may be recommended [19, 21]. In terms of OPMD, the intraoral manifestations of OLP generally include symmetrical white striae interspersed with irregular erosions [23, 24]. Discoid lupus erythematosus may be characterised by erosions surrounded by radial white striae, commonly seen in the lower lip [26]. Oral leucoplakia, or oral erythroplakia, is primarily characterised by white or scarlet patches accompanied by persistent erosions, and histological examination is essential for the ultimate diagnosis of OPMD [25, 33]. In addition, oral malignant lesions, predominantly represented by OSCC, tend to present as localized ulcers with a firm texture on their margins upon palpation. The tongue is the most frequently involved site for these ulcers [14]. Based on the features of the pathological examination and blood test results, all of the above diagnoses could be excluded for this patient.

Additionally, some relatively rare diseases may mimic the form of multiple intraoral persistent erosions, including paraneoplastic pemphigus (PNP) or PAMS [9], oral infections such as TB [22], haematopoietic and lymphoid neoplasms including Langerhans cell histiocytosis (LCH) [29], genetic diseases represented by dyskeratosis congenita [8], and pyostomatitis vegetans (PSV) [34]. PNP, also known as PAMS, is a lethal autoimmune disease associated with tumours such as thymoma. Because multiple organs may be involved, the term PAMS has been proposed to fully reflect the clinical manifestations and immunopathological features of the disease [9, 18, 35]. Clinically, PAMS manifests as erosions or bulla of the skin, and mucous membranes might be demonstrated [12]. In line with pemphigus, the diagnosis of PAMS also relies on HE and DIF [12, 36]. Therefore, a diagnosis of PAMS was excluded. Oral TB can also manifest as recalcitrant ulcers or erosions with irregular margins, for which typical Langerhans giant cells viewed under the microscope and the presence of TB DNA upon quantitative polymerase chain reaction (qPCR) are the dominant features [3, 22]; therefore, oral TB was eliminated from this case. Haematopoietic and lymphoid malignancies containing LCH might also be characterised by erosions [30]. HE, IHC, and blood tests are important clues for diagnosis. Additionally, genetic diseases such as dyskeratosis congenita can also show similar features [8], for which whole-exome sequencing is an indispensable diagnostic marker, in addition to biopsy of the lesion. Moreover, PSV, as a relatively specific sign of inflammatory bowel disease (IBD), can also present as proliferative pustular lesions and subsequent erosions in the oral mucosa [27], the diagnosis of which largely depends on clinical presentation, peripheral eosinophilia, and histological characteristics [28]. Notably, proliferative lesions displaying nodule-like or granular-like forms might be observed in the erosive lesions of oral diseases such as oral TB [22], syphilis [20, 37], pemphigus vegetans [38], lymphoma [39], OSCC [40], and PSV [41]; therefore, these diseases cannot be ignored as potential diagnoses when oral erosions are accompanied by proliferative lesions.

Based on the features of the aforementioned diseases and the systemic findings, the oral presentations of the patient and differential diagnosis were made, and the ultimate conclusion was oral manifestations of FL, one of the most frequent NHL. In Western countries, FL accounts for approximately 5% of all haematologic neoplasms and about 20–25% of all newly diagnosed NHL [31]. Up to 40% of NHL were identified at extranodal sites, of which the head and neck region is the second most involved site of extra-nodal lymphomas [42]. However, primary lymphomas of the oral cavity are very rare, accounting for only 3% of all lymphomas in the general population [43]. According to a study by Barone S et al., the most commonly involved sites are in the soft tissues of oral cavity, with an incidence rate presenting as 38.4%, 19.2%, and 11.5% for buccal mucosa, tongue and gingiva [44]. As for the clinical appearance, lymphoma occurring in the gingiva, buccal mucosa, and palate usually manifests as ulceration (Table 2) [39, 45–56], while that of FL has been discussed above, and masses or nodular lesions may be observed or palpated under the mucosa of the palate, buccal, and tongue [39, 45, 50, 51, 57–60]. Swelling of the lip, palate, gingiva, or buccal mucosa may serve as alternative oral signs of lymphoma [61–64]. Meanwhile, accompanying symptoms such as enlargement of multiple superficial lymph nodes in the head and neck region and mucosal necrosis have also been reported [47]. To the best of our knowledge, this is the first report of oral manifestations of FL exhibiting widespread erosion interspersed with proliferative lesions in the oral mucosa. However, it seems difficult to judge whether the initial site of manifestation of the FL was in the oral mucosa or in the retroperitoneal area, based on the medical history of this case.

**Table 2:** Summarized oral manifestations of lymphomas reported in previous literatures

Author-year, country	Diagnosis of lymphoma	Oral mucosal manifestations
**Campeanu AT, et al.-2022, Romania** [55]	Plasmablastic lymphoma	A large mass with ulcerated surface located on the left mandible region
**Hafian H, et al.-2021, Japan** [57]	Oral mucosa-associated lymphoid tissue lymphoma	An invisible irregular submucosal nodular lesion in the right buccal
**Lyu X, Guan X-2021, China** [56]	Extranodal natural killer T-cell lymphoma (nasal type)	Hard palate ulceration with irregular border between top two front teeth
**Coskunses FM, et al.-2020, Turkey** [45]	Diffuse large B-cell lymphomas	Gingival erythema and buccal swelling with no ulceration or suppuration. Regular shaped mass of buccal mucosa
**Yu W, Park C, Shimel B-2020, United States of America** [58]	Double-hit B-cell lymphoma	A large soft mass with purplish-red and grossly surface, intact without obvious ulceration, expansile lesion of the left maxillary alveolus
**de Andrade BAB, et al.-2020, Brazil** [39]	Anaplastic large cell lymphoma	Swelling of posterior alveolar ridge of the left maxilla, covered by ulcerated mucosa
**de Andrade BAB, et al.-2020, Brazil** [39]	Anaplastic large cell lymphoma	An ulcerated mass located at the floor of the mouth
**de Andrade BAB, et al.-2020, Brazil** [39]	Anaplastic large cell lymphoma	A mass with irregular surface on the posterior alveolar ridge of the right mandible
**de Andrade BAB, et al.-2020, Brazil** [39]	Anaplastic large cell lymphoma	An ulcerated red mass with irregular surface on the hard palate
**Kamat M, et al.-2019, India** [59]	Burkitt's lymphoma	A sessile exophytic mass covered by slough at left posterior mandible region
**Batta N, et al.-2019, India** [47]	Diffuse large B-cell lymphomas	Grayish-brown ulcerative growth on the buccal mucosa and palpable lymph nodes in the right neck
**Marcucci M, et al.-2017, Brazil** [60]	Mantle cell lymphoma	A tumor mass on the left side of the floor of the mouth
**Booken N, et al.-2013, Germany** [46]	Lymphomatoid papulosis, type A	Ulcerated nodules on the oral mucosa
**Kämmerer PW, et al.-2013, Germany** [48]	Hodgkin’s lymphoma	Ulcerating lesion of the left retromolar region of the mandible
**Frei M, et al.-2012, Switzerland** [61]	Diffuse large B-cell lymphomas	Diffuse swelling of the buccal mucosa and palate
**Patil K, Mahima VG, Srikanth HS-2010, India** [64]	Non-Hodgkin’s lymphoma	Multinodular swelling on the buccal gingiva
**Mignogna MD, et al.-2009, Italy** [63]	Diffuse large B-cell lymphomas	Diffuse micropapillary lesions on the hard palate and inner upper lips, “cerebriform” aspect of the right cheek
**Niscola P, et al.-2009, Italy** [49]	Mucosa-associated lymphoid tissue lymphoma	Ulcerative lesion on the lip
**Wain EM, et al.-2003, United Kingdom** [50]	Mycosis fungoides	Asymptomatic thickened spongy lesion with ulceration on the soft palate, hyperplasia with ulceration on the lateral border of the tongue and thickening of the lip
**Hata T, et al.-1998, Japan** [51]	Mycosis fungoides	Erythematous, ulcerated, necrotic mass with irregular margins and covered by white patches on the gingiva and the buccal mucosa
**Vicente A, et al.-1991, Spain** [52]	Mycosis fungoides	Erythematous, nonulcerated plaque on the hard palate, and erythema, induration, together with ulceration of the gingiva
**Vicente A, et al.-1991, Spain** [52]	Mycosis fungoides	Indurated and ulcerated plaque, covered by a grayish-white pseudomembrane on the hard palate
**Zanakis SN, et al.-1992, Greece** [62]	Non-Hodgkin’s lymphoma	Firm swelling of the whole cheek
**Chuong R, et al.-1984, United States of America** [53]	Diffuse, mixed lymphocytic, and histiocytic lymphoma	Ulcer with white pseudomembrane involving the posterior portion of the right maxilla and left soft palate
**Yokobayashi Y, et al.-1981, Japan** [54]	Malignant mesenchymal tumor (possibly malignant lymphoma of reticulum cell type)	Ulcer with an indurated margin of the buccal mucosa, gingiva, maxillary tuberosity and soft and hard palate

The pathogenesis of FL that leads to oral mucosal erosion and proliferative lesions is unknown. However, this process may be associated with the tumour immune microenvironment. In FL, the tumour microenvironment is one of the main contributors to tumour cell survival and proliferation. These important components of the microenvironment, such as follicular germinal centers, helper T cells, macrophages, and FDCs, play crucial roles in the formation of oral FL lesions [65]. In FL with poor prognosis, the functional genes of FDC were relatively overexpressed, and the helper T cells were functionally active, producing various cytokines such as IL-2, IL-12, and IFN-γ [66]. Thus, the abnormal activity of these immune cells and overexpression of cytokines may be responsible for the onset of oral mucosal lesions.

**Fig. 4:**
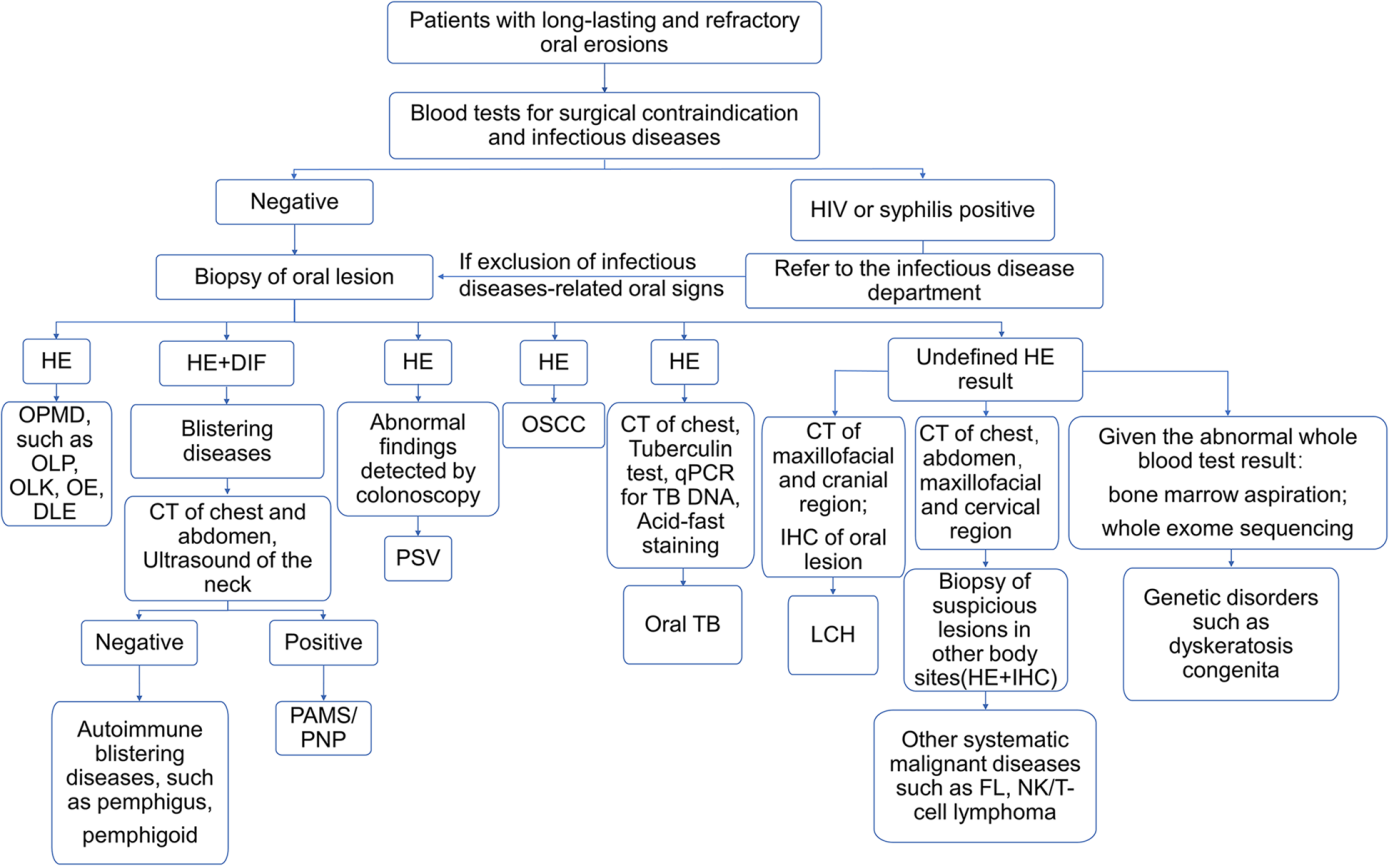
Diagnostic algorithms for diseases characterized by refractory erosion of the oral mucosa proposed by the authors of this work. Abbreviations: HIV: human immunodeficiency virus; HE: hematoxylin–eosin; OPMD: oral potentially malignant disorders; OLP: oral lichen planus; OLK: oral leukoplakia; OE: oral erythroplakia; DLE: discoid lupus erythematosus; DIF: direct immunofluorescence; CT: computed tomography; PAMS: paraneoplastic autoimmune multiorgan syndrome; PNP: paraneoplastic pemphigus; PSV: pyostomatitis vegetans; OSCC: oral squamous cell carcinoma; qPCR: quantitative polymerase chain reaction; TB: tuberculosis; LCH: Langerhans cell histiocytosis; IHC: immunohistochemistry; FL: follicular lymphoma

Based on the diagnostic process of this particular case and the logistics of differential diagnosis, we have summarised a flowchart of the diagnostic algorithm for patients with refractory oral erosions (Fig. 4), thus ensuring a fast and accurate diagnosis for patients presenting in oral medicine clinics.

The clinical manifestations of intraoral FL are mostly masses or swelling of the oral mucosa [32]. This case serves as the initial report of FL presenting as scattered erosions and proliferative lesions in the oral mucosa. Upon receiving patients with widespread and refractory oral mucosal erosions, the common diagnosis of OLP, autoimmune bullous disease, HIV, and syphilis should be initially considered, and PAMS, oral TB, LCH, genetic diseases, and PSV should also be evaluated. If all these are excluded, accompanied by the ineffectiveness of the conventional treatment, clinicians should be aware of systemic malignancies such as FL. 

## Data Availability

Not applicable.

## Abbreviations

AIDS: Acquired immune deficiency syndrome
BCL-2: B-cell lymphoma 2
CT: Computed tomography
DIF: Direct immunofluorescence
DLE: Discoid lupus erythematosus
FDC: Follicular dendritic cell
FL: Follicular lymphoma
FLIPI: Follicular lymphoma international prognostic index
HE: Hematoxylin–eosin
HIV: Human immunodeficiency virus
IBD: Inflammatory bowel disease
IHC: Immunohistochemistry
LCH: Langerhans cell histiocytosis
NHL: Non-Hodgkin’s lymphomas
OE: Oral erythroplakia
OLK: Oral leukoplakia
OLP: Oral lichen planus
OPMD: Oral potentially malignant disorders
OSCC: Oral squamous cell carcinoma
PAMS: Paraneoplastic autoimmune multiorgan syndrome
PET/CT: Positron emission tomography/computed tomography
PNP: Paraneoplastic pemphigus
PSV: Pyostomatitis vegetans
qPCR: Quantitative polymerase chain reaction
RAU: Recurrent aphthous ulcer
R-CHOP: Rituximab plus cyclophosphamide, doxorubicin, vincristine, and prednisone
TB: Tuberculosis
